# Supplementary material for: Molecular architecture determines brain delivery of a transferrin receptor–targeted lysosomal enzyme
Source: J Exp Med. 2022 Feb 26;219(3):e20211057. doi: 10.1084/jem.20211057 (PMC8932535; doi:10.1084/jem.20211057)
Supplement: Table S1 — lists PK parameters. [file JEM_20211057_TableS1.docx]

**A**

|  | Drug | Dose  (mg/kg) | ^†^T_max_  (h) | ^††^C_0_ or C_max_  (nM) | AUC_0-last_  (nM*h) | Vss (L/kg) | CL (mL/h/kg) |
| --- | --- | --- | --- | --- | --- | --- | --- |
| Serum | ETV:IDS  IgG:IDS | 1 | -  - | 174  22.4 | 935  - | 0.0976  - | 9.03  - |
|  | ETV:IDS  IgG:IDS | 3 | -  - | 635  345 | 3940  408 | 0.0600  0.130 | 6.80  58.2 |
|  | ETV:IDS  IgG:IDS | 10 | -  - | 1970  1390 | 15300  3040 | 0.0482  0.0646 | 5.63  26.1 |
| Brain | ETV:IDS  IgG:IDS | 1 | 8  24 | 3.43  3.25 | 68  59.1 | -  - | -  - |
|  | ETV:IDS  IgG:IDS | 3 | 8 (8 - 24)  8 | 6.79  3.91 | 134  84.1 | -  - | -  - |
|  | ETV:IDS  IgG:IDS | 10 | 24 (8 - 24)  8 (8 - 24) | 12  4.50 | 229  92 | -  - | -  - |
| Liver | ETV:IDS  IgG:IDS | 1 | 8  4 (0.5 - 4) | 48.3  251 | 893  4020 | -  - | -  - |
|  | ETV:IDS  IgG:IDS | 3 | 4 (4 - 8)  4 | 93.7  530 | 1730  7430 | -  - | -  - |
|  | ETV:IDS  IgG:IDS | 10 | 6 (4 - 8)  2.25 (0.5 – 4) | 187  1100 | 3260  14100 | -  - | -  - |

**B**

|  | Drug | Dose  (mg/kg) | 0.5 hour | 4 hour | 8 hour | 24 hour |
| --- | --- | --- | --- | --- | --- | --- |
| Serum concentration [nM] ± SD | ETV:IDS  IgG:IDS | 1 | 154 ± 55  15.7 ± 4.8 | 65.6 ± 27  1.58 ± 0.77 | 33.2 ± 10  0.79 | 9.36 ± 1.7  BQL |
|  | ETV:IDS  IgG:IDS | 3 | 578 ± 35  218 ± 90 | 301 ± 36  10.5 ± 5.8 | 137 ± 54  2.15 ± 0.83 | 27.0 ± 7.6  0.38 ± 0.17 |
|  | ETV:IDS  IgG:IDS | 10 | 1820 ± 390  1090 ± 280 | 1050 ± 170  209 ± 68 | 849 ± 240  32.2 ± 16 | 114 ± 37  0.97 ± 0.31 |
| Brain  concentration [nM] ± SD | ETV:IDS  IgG:IDS | 1 | 1.51 ± 0.15  1.75 ± 0.49 | 2.53 ± 0.49  2.33 ± 0.94 | 3.43 ± 0.57  2.09 ± 0.93 | 2.69 ± 0.23  3.25 ± 0.63 |
|  | ETV:IDS  IgG:IDS | 3 | 2.44 ± 0.26  2.19 ± 0.15 | 4.91 ± 0.67  3.15 ± 0.45 | 6.37 ± 1.3  3.91 ± 0.32 | 5.87 ± 1.3  3.61 ± 0.22 |
|  | ETV:IDS  IgG:IDS | 10 | 3.80 ± 0.68  2.43 ± 0.24 | 6.16 ± 0.55  3.55 ± 1.1 | 11.7 ± 2.4  4.72 ± 0.98 | 11.5 ± 1.9  3.69 ± 0.48 |
| Liver  concentration [nM] ± SD | ETV:IDS  IgG:IDS | 1 | 31.7 ± 5.6  150 ± 80 | 38.4 ± 6.4  250 ± 42 | 48.3 ± 13  193 ± 27 | 27.5 ± 8.0  114 ± 36 |
|  | ETV:IDS  IgG:IDS | 3 | 57.5 ± 9.8  421 ± 140 | 84.0 ± 19  530 ± 46 | 88.3 ± 16  343 ± 7.4 | 54.7 ± 13  170 ± 23 |
|  | ETV:IDS  IgG:IDS | 10 | 102 ± 50  958 ± 330 | 162 ± 28  1030 ± 180 | 186 ± 48  738 ± 73 | 92.3 ± 13  232 ± 24 |

**Supplemental Table 1. Pharmacokinetic parameters. (A)** Serum, brain, and liver pharmacokinetic parameters at 1, 3, and 10 mg/kg of ETV:IDS or IgG:IDS. ^†^T_max_ is median (range) in brain and liver. ^††^C_0_ is indicated for serum whereas C_max_ is indicated for brain and liver. CL = serum clearance; Vss = steady-state distribution volume. **(B)** Serum, brain and liver concentrations of ETV:IDS or IgG:IDS from TfR^mu/hu^ KI mice were measured 0.5, 4, 8, and 24 hours after an intravenous dose of 1, 3 or 10 mg/kg and determined using an IDS capture/IDS detect immunoassay. TfR^mu/hu^ KI mice are on a C57BL/6 background; n = 3-5 per group. Table displays mean values ± SD. BQL = below quantitation limit (Lower limit of quantitation = 0.00412 nM).
