# Supplementary material for: Molecular architecture determines brain delivery of a transferrin receptor–targeted lysosomal enzyme
Source: J Exp Med. 2022 Feb 26;219(3):e20211057. doi: 10.1084/jem.20211057 (PMC8932535; doi:10.1084/jem.20211057)
Supplement: Table S2 — shows that ETV:IDS is more effective than IgG:IDS at reducing brain and CSF GAGs in Ids KO;TfRmu/hu KI mice. [file JEM_20211057_TableS2.docx]

**A**

Fold over TfR^mu/hu^ KI GAG levels in the Liver, CSF, and Brain

|  | Liver | | | CSF | | Brain | |
| --- | --- | --- | --- | --- | --- | --- | --- |
| Dose  (mg/kg) | ETV:IDS | IgG:IDS | | ETV:IDS | ­IgG:IDS | ETV:IDS | IgG:IDS |
| 0 | 161.3 | | | 44.1 | | 28.7 | |
| 1 | 10.5 | | 9.9 | 14.1 | 29.8 | 14.6 | 21.6 |
| 3 | 7.4 | | 6.4 | 11.4 | 18.4 | 12.2 | 20.0 |
| 10 | 5.6 | | 5.2 | 8.9 | 17.2 | 6.8 | 16.4 |

**B**

Percent Reduction from *Ids* KO;TfR^mu/hu^ KI + Vehicle GAG levels in the Liver, CSF, and Brain

|  | Liver | | | CSF | | Brain | |
| --- | --- | --- | --- | --- | --- | --- | --- |
| Dose  (mg/kg) | ETV:IDS | IgG:IDS | | ETV:IDS | ­IgG:IDS | ETV:IDS | IgG:IDS |
| 1 | 93.5 | | 93.9 | 68.0 | 32.4 | 49.0 | 24.7 |
| 3 | 95.4 | | 96.0 | 74.1 | 58.3 | 57.4 | 30.4 |
| 10 | 96.5 | | 96.8 | 80.0 | 61.0 | 76.2 | 42.7 |

**Supplemental Table 2.** **ETV:IDS is more effective than IgG:IDS at reducing brain and CSF GAGs in *Ids* KO;TfR^mu/hu^ KI mice.** GAG levels were evaluated in the liver, CSF and brain of *Ids* KO;TfR^mu/hu^ KI mice 7 days following treatment with ETV:IDS or IgG:IDS after an intravenous dose of 1, 3, or 10 mg/kg and compared to vehicle treatment and non-diseased littermate control TfR^mu/hu^ KI mice. GAG values calculated include **(A)** Fold over TfR^mu/hu^ KI and **(B)** Percent reduction from vehicle treated *Ids* KO;TfR^mu/hu^ KI mice. All mice are on a C57BL/6 background; n = 5 per group.
